# Molecular Detection of *Blastocystis* Subtypes ST7 and ST26 in Fecal Samples from Black-Headed Gulls in Kunming, China

**DOI:** 10.3390/vetsci13070685

**Published:** 2026-07-14

**Authors:** Jianliang Zhang, Penghao Wei, Yidan Wang, Zhiyan Sun, Junjun He, Feiyan Dai, Jianfa Yang, Fengcai Zou, Luyang Wang

**Affiliations:** 1 The Yunnan Key Laboratory of Veterinary Etiological Biology, College of Veterinary Medicine, Yunnan Agricultural University, Kunming 650201, China; 2 College of Veterinary Medicine, Yunnan Agricultural University, Kunming 650201, China

**Keywords:** black-headed gull, *Blastocystis*, sample positivity, subtype, zoonotic transmission

## Abstract

Migratory birds are potential carriers for the cross-regional spread of zoonotic diseases due to their high mobility and extensive geographical activity range. *Blastocystis*, a ubiquitous parasitic protist, can infect a broad spectrum of animal hosts including humans, constituting a potential public health hazard. Currently, data on *Blastocystis* infection in black-headed gulls (*Chroicocephalus ridibundus*) remain scarce. This study aimed to investigate the fecal sample positivity rate, epidemiological characteristics, and subtype distribution of *Blastocystis* in wintering black-headed gulls in Kunming, China. A total of 245 fresh fecal samples were collected from black-headed gulls in Kunming, Yunnan Province, during December 2025 and January 2026. Fecal DNA was extracted and subjected to PCR amplification targeting the small subunit ribosomal RNA (*SSU* rRNA) gene of *Blastocystis*. Results showed a 3.27% (8/245) overall *Blastocystis* fecal sample positivity rate in wintering black-headed gulls, with two subtypes detected: dominant zoonotic pathogenic ST7 (87.5%, 7/8) and ruminant-adapted ST26 (12.5%, 1/8). This study represents the first report of *Blastocystis* in black-headed gulls in China, providing data supporting the understanding of genetic subtype diversity of *Blastocystis* in avian hosts.

## Simple Summary

*Blastocystis* is a zoonotic parasitic microorganism that infects both humans and animals, posing persistent threats to public health. Migratory birds possess long-distance migration characteristics and wide activity ranges, enabling them to carry and disseminate various pathogens across regions. This study investigated *Blastocystis* positivity in fecal samples in wintering black-headed gulls (*Chroicocephalus ridibundus*) in Kunming, China. A total of 245 fresh fecal samples were collected, and DNA was extracted and tested using PCR targeting the *SSU* rRNA gene of *Blastocystis*. The results showed that 3.27% (8/245) of the samples were positive for *Blastocystis*. Among these, two subtypes were identified: ST7 (87.5%, 7/8) and ST26 (12.5%, 1/8). ST7 is a zoonotic pathogenic subtype commonly found in birds and humans, while ST26 has been primarily reported in ruminants and is here reported for the first time in black-headed gulls worldwide. These findings suggest that black-headed gulls may serve as potential carriers of *Blastocystis* and could contribute to its cross-species transmission, emphasizing the necessity of long-term pathogen surveillance of migratory birds for public health prevention and control.

## 1. Introduction

*Blastocystis* is an obligate anaerobic parasitic protist commonly found in the gastrointestinal tract of humans and a wide variety of animal species, demonstrating extensive host adaptability [1]. It has been reported in over 200 distinct host species, including humans, birds, amphibians, reptiles, and even arthropods [2,3,4]. Transmission occurs primarily via the fecal-oral route, with infection resulting from the ingestion of water or food contaminated with environmentally resistant cysts [5]. Recent epidemiological surveys indicate that *Blastocystis* is the most frequently detected unicellular eukaryotic organism in human fecal samples, and an estimated 1–2 billion people worldwide harbour this protist, with elevated colonization rates prevalent among children and immunocompromised populations [4,6]. The clinical manifestations of *Blastocystis* infection range from mild, self-limiting abdominal discomfort to chronic persistent diarrhea and cutaneous lesions [4]. The severity and clinical presentation depend on the infecting subtype [7,8].

Based on the analysis of sequence polymorphisms in the small subunit ribosomal DNA (*SSU* rDNA) gene, *Blastocystis* exhibits high genetic diversity, with 45 subtypes (STs) having been identified to date [5,9]. Among these, ST1 through ST9 and ST12 have been detected in humans, with ST1–ST4 being the most prevalent and exhibiting cross-species transmission potential [10]. ST5 is frequently found in perissodactyl and artiodactyl animals (e.g., pigs) [11], ST6 and ST7 are commonly detected in birds and poultry, and ST8 is often identified in non-human primates [12]. In contrast, ST10 and ST14 appear to be host-specific, having been identified only in certain animals such as cattle and sheep [13]. Mixed infections with multiple *Blastocystis* subtypes have been documented in various animal species [14]. The evolutionary diversity of *Blastocystis* subtypes arises from inter-subtype genetic recombination and transmission across different host species.

Debates surrounding the pathogenic capability of *Blastocystis* have persisted for years, largely because the majority of infected individuals remain asymptomatic. However, relevant studies have reported that *Blastocystis* was the only detectable potential pathogen in pediatric patients with diarrhea. The affected child achieved full recovery after antiparasitic treatment, with subsequent tests returning negative. Meanwhile, 26 family members of the patient developed gastroenteritis simultaneously. These findings indicate that *Blastocystis* has pathogenic potential and can trigger clustered outbreaks via shared contaminated water or person-to-person transmission [15]. Moreover, accumulating in vitro and in vivo evidence supports the pathogenic potential of *Blastocystis*, with pathogenicity being subtype-dependent [7,8,16]. For example, the zoonotic avian ST7 subtype strongly adheres to Caco-2 cells at intercellular junctions. Disruption of tight junctions by this subtype compromises intestinal barrier function and increases transepithelial permeability [17,18]. In addition, existing studies have revealed that ST7 infection exerts pathogenic effects via disrupting the intestinal microbiota, reducing gut microbial diversity in the host and ultimately inducing intestinal dysbiosis [7,19].

Among the various animal hosts of *Blastocystis*, birds have historically received insufficient attention. However, recent molecular epidemiological investigations have revealed that birds harbor a wide range of *Blastocystis* subtypes and serve as the primary natural reservoir for the avian-adapted ST6 and ST7 [12]. Of particular concern are wild migratory birds, especially waterfowl, which migrate long distances across different countries and habitats. Their ability to traverse geographic regions and ecosystems via annual migrations along major global flyways—between breeding, stopover, and wintering grounds-may play a unique role in the cross-regional dissemination of *Blastocystis*, posing a potential public health threat [20,21,22]. Despite the wealth of epidemiological data on *Blastocystis* in humans and domestic animals, information on *Blastocystis* in wild migratory birds remains limited. Recent studies have reported a global prevalence of 26.4% for *Blastocystis* in birds, with 19 subtypes identified (ST1–ST10, ST14, ST20, ST21, ST23–ST25, ST27–ST29), of which 12 (ST1–ST10, ST14, ST23) are considered to have zoonotic potential [23].

Kunming, the capital of Yunnan Province in southwestern China, serves as a core wintering ground for black-headed gulls. From October to November each year, large numbers of black-headed gulls migrate to Kunming for overwintering and usually remain there until March of the following year. Previous tracking and observational studies have indicated that the wintering population in Kunming mainly originates from northern breeding areas, including the Lake Baikal region of Russia/Siberia, Mongolia, and parts of northwestern China, before migrating southward to Dianchi Lake and other urban wetland habitats in Kunming. During the overwintering period, hundreds of thousands of tourists visit Dianchi Lake and other locations to observe and feed the gulls, resulting in frequent and close human–gull contact [24,25].

In this setting, frequent human–gull contact may increase opportunities for environmental exposure to Blastocystis cysts, although actual zoonotic transmission has not been confirmed. This study aimed to elucidate the fecal sample positivity rate of *Blastocystis* in black-headed gulls, fill the research gap concerning *Blastocystis* in this bird species in China, contribute to the broader investigation of *Blastocystis* infection in wild birds, and provide data to support public health risk assessment.

## 2. Materials and Methods

### 2.1. Ethical Statement

The research protocol was reviewed and approved by the Research Ethics Committee of Yunnan Agricultural University. Sample collection was performed in compliance with the relevant provisions of the Regulations for the Implementation of the Law of the People’s Republic of China on the Protection of Terrestrial Wildlife and the Wildlife Protection Law of the People’s Republic of China. No harm was caused to migratory black-headed gulls during the sampling process.

### 2.2. Sample Collection

**Figure 1:**
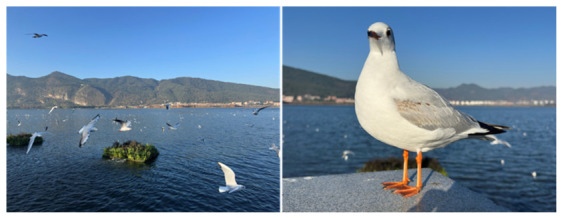
Migratory Black-Headed Gulls (*Chroicocephalus ridibundus*) at the Dianchi Lake in Kunming, Yunnan province, China.

A total of 245 fresh fecal samples from wintering black-headed gulls were collected in Kunming City, Yunnan Province, China, during December 2025 (*n* = 88) and January 2026 (*n* = 157) (Figure 1 and Table 1). Fresh fecal samples were collected immediately after direct observation of defecation by black-headed gulls. Samples were collected from the ground using sterile disposable tools, and only freshly deposited feces clearly attributable to black-headed gulls were included. Information was recorded during sampling. The samples were kept refrigerated during transport, immediately transferred to the laboratory upon return, and stored at 4 °C for subsequent use.

### 2.3. DNA Extraction

Approximately 200–300 mg of each fecal sample was placed into a centrifuge tube. Fecal whole genomic DNA extraction was performed using the E.Z.N.A.^®^ Stool DNA Kit (Omega Bio-Tek, Norcross, GA, USA). The quality of the DNA samples was assessed using Nanodrop 2000 (Thermo Fisher Scientific, Waltham, MA, USA), and high–quality extracted samples were then labeled, sub–packaged into 200 μL sterile centrifuge tubes, and stored at −20 °C.

### 2.4. PCR Amplification

*Blastocystis* in all samples was detected using nested-PCR amplification of the partial *SSU* rRNA gene. The nested PCR employed external primers RD5 (5′-GGAAGCTTATCTGGTTGATCCTGCCAGTA-3′) and RD3 (5′-GGGATCCTGATCCTTCCGCAGGTTCACCTAC-3′), as well as internal primers Bla1 (5′-GGAGGTAGTGACAATAAATC-3′) and Bla2 (5′-TGCTTTCGCACTTGTTCATC-3′). The internal primer set produced a 479-bp amplicon [26,27]. PCR assays included positive and negative controls and all samples were tested in triplicate. The PCR products were mixed with DNA Green reagent (Tiandz, Inc., Beijing, China) for staining (1:5), and then detected by 1.0% agarose gel electrophoresis for the presence of the target fragment in the PCR amplification products. Positive PCR products were sent to Sangon Biotech (Shanghai, China) for sequencing.

### 2.5. Sequence Analysis and Subtype Identification

The raw sequencing data were edited and assembled using SeqMan software（v7.0） within the DNAStar package. The assembled sequences were submitted to the NCBI GenBank database for BLAST analysis (http://www.ncbi.nlm.nih.gov/BLAST/, accessed on 2 June 2026) and identified by alignment with a highly homologous reference sequence to identify *Blastocystis* sp. The phylogenetic evolutionary tree was constructed using the neighbor-joining method in MEGA 12 (http://www.megasoftware.net, accessed on 3 June 2026) with 1000 bootstrap replicates.

### 2.6. Statistical Analysis

The fecal sample positivity rate of Blastocystis and its 95% confidence interval (CI) were calculated. Differences between sampling months were analyzed using Fisher’s exact test in IBM SPSS Statistics 20.0 (IBM Corp., Armonk, NY, USA). A value of *p* < 0.05 was considered statistically significant.

## 3. Results

### 3.1. PCR Amplification Results and Blastocystis Sample Positivity

The PCR amplification (positive) results of *SSU* rRNA of *Blastocystis* sp. produced bands of approximately 479 bp, consistent with the expected fragment size. PCR amplification and sequencing revealed that 8 of the 245 fecal samples were positive for *Blastocystis*, yielding an overall detection rate of 3.27% (8/245) in wintering black-headed gulls at Dianchi Lake, Kunming City, Yunnan Province. As shown in Table 1, there was no significant difference in *Blastocystis* fecal sample positivity rate between December and January (*p* > 0.05).

### 3.2. Distribution of Blastocystis Subtypes

In the present study, two *Blastocystis* subtypes were recovered, namely ST7 (n = 7) and ST26 (n = 1). Among the eight positive samples, ST7 was the most frequently detected subtype, while ST26 was detected in only one sample. Although ST26 is a subtype prevalent in ruminants, it was discovered for the first time in fecal samples of black-headed gulls in our study, with just one positive case. In the 88 total samples collected in December 2025, only ST7 (n = 3) was present, while both ST7 (n = 4) and ST26 (n = 1) were detected across 157 samples from January (Table 1). And no mixed subtypes infection was found in the present research.

### 3.3. Phylogenetic Analysis of Blastocystis sp.

**Figure 2:**
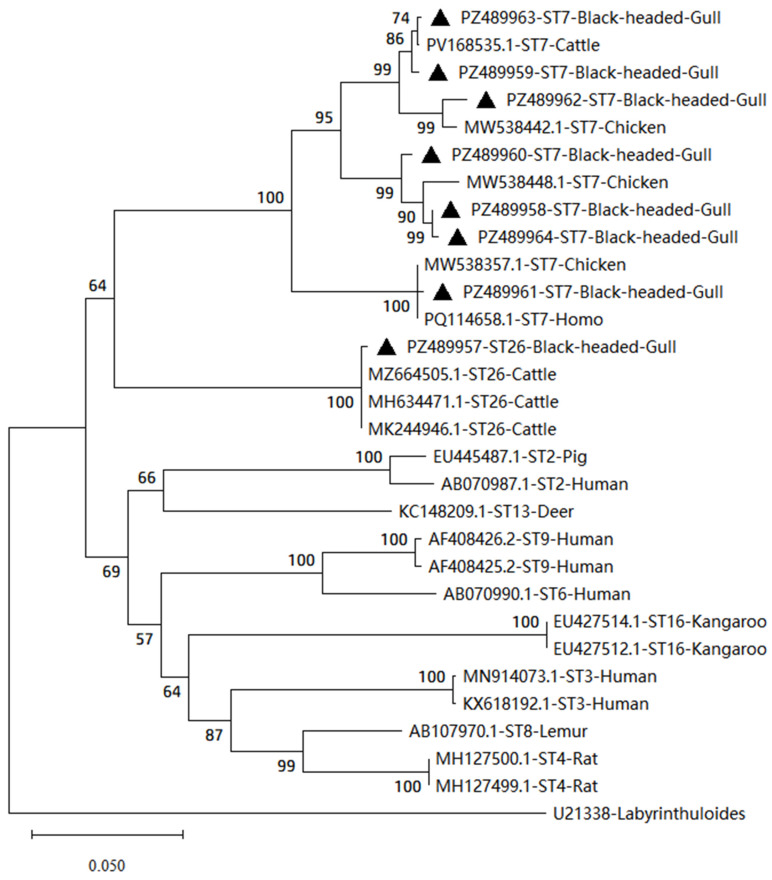
Phylogenetic tree based on the *SSU* rRNA gene of *Blastocystis*. The ▲ indicate the sequences acquired from the current study.The scale bar at the bottom (0.050) indicates the genetic distance.

In our study, we sequenced 8 positive isolates and obtained 8 representative sequences. The sequences obtained are highly homologous to the reference sequence of *Blastocystis* sp. in the GenBank database (Figure 2). A phylogenetic tree was constructed based on the *SSU* rRNA gene of *Blastocystis* using the neighbor-joining method in MEGA 12.0, with *Labyrinthuloides haliotidis* as the outgroup. Nucleotide sequence alignment revealed that the ST7 genotypes detected in this study shared 98.36% to 99.79% identity with the published reference sequences (GenBank Accession Nos. PV168535, PQ114658, MW538442, MW538448, MW538357). Phylogenetic reconstruction further confirmed that all the obtained ST7 sequences formed an independent monophyletic clade together with previously reported ST7 isolates derived from humans, avian hosts and cattle. Notably, the ST26 sequence identified in this study showed 100% sequence identity with bovine-derived ST26 isolate sequences (Accession Nos. MZ664505, MH654471, MK244946), separate from the clusters of typically bird-adapted subtypes.

## 4. Discussion

In this study, *Blastocystis* was detected in 8 of 245 fecal samples collected from black-headed gulls, yielding a positivity rate of 3.27%. Compared with the migratory birds or other bird species, the positivity rate of *Blastocystis* in black-headed gulls was relatively low. So far, *Blastocystis* infection in birds has been reported in various regions in China. For example, in overwintering migratory whooper swans (*Cygnus cygnus*) in the Sanmenxia Wetland of Henan Province, the positivity rate was 11.6% [20]; in peafowls (*Pavo cristatus*) in Henan Province, the positivity rate was 35% [28]; in common quails (*Coturnix coturnix)* in Xinxiang City, Henan Province, the positivity rate was 7.8% [29]; and in red-crowned cranes (*Grus japonensis*) in Heilongjiang Province, the positivity rate was 14% [30]. Previous studies have reported an overall *Blastocystis* positivity rate of 5.9% in migratory bar-headed geese across China. Regionally, the positivity rate was 5.0% in Aba of Sichuan, 11.8% in Maqu of Gansu, and 0.8% in Caohai Wetland of Weining, Guizhou [21]. In Sichuan Province, *Blastocystis* infection has been confirmed in multiple bird species, with positivity rates of 18.2% in ruddy shelducks (*Tadorna ferruginea*), 10.5% in black swans (*Cygnus atratus*), and 6.7% in green peafowls (*Pavo muticus*) [31]. This difference is likely attributable to the distinct habitat and feeding ecology of black-headed gulls. As typical migratory waterbirds that primarily inhabit lakes, rivers, wetlands, and coastal waters, black-headed gulls mainly feed on aquatic organisms such as small fish, shrimp, and plankton, and rarely consume soil humus, soil insects, or contaminated vegetation. This dietary habit substantially reduces their exposure to soil-borne and fecal-borne parasite eggs and protozoan cysts, which may explain the lower detection rate observed in this study compared with most terrestrial and free-range bird species. Nevertheless, since relevant variables such as dietary composition, habitat type, exposure to contaminated water or sediment, environmental contamination level, contact with domestic animals or humans, and individual host factors such as age, sex, and health status were not explored in this study, the above explanation remains hypothetical, and further targeted investigations are therefore required.

**Figure 3:**
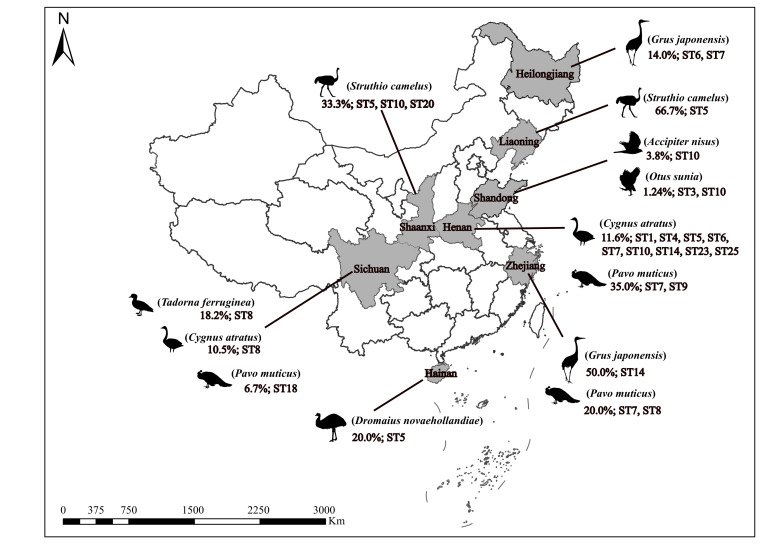
Prevalence and subtype distribution of *Blastocystis* in wild birds in China.

A total of fourteen subtypes (ST1, ST3, ST4, ST5, ST6, ST7, ST8, ST9, ST10, ST14, ST18, ST20, ST23, and ST25) of *Blastocystis* have been detected in wild birds in China (Figure 3). In our study, ST7 and ST26 were detected in the black-headed gulls. To our knowledge, this is the first research to report on the occurrence of subtypes ST7 and ST26 in the black-headed gulls. Although the number of positive samples was limited, the frequent detection of ST7 among the positive samples is consistent with previous reports that this subtype is frequently identified as a bird-adapted subtype across various geographical regions including Brazil [32], Thailand [33], Malaysia [34], and other parts of China [20,21,29]. Notably, ST7 is a zoonotic pathogenic subtype [17,18,19]. Black-headed gulls are migratory waterbirds characterized by wide-ranging activity, cross-regional migration, and flocking behavior. The ST7 subtype detected in these gulls may be released into the environment through fecal excretion of cysts, potentially contaminating wetland water bodies, mudflats, and surrounding areas. This finding suggests that black-headed gulls could participate in environmental circulation of Blastocystis. However, given the low sample positivity rate observed in this study, their role in cross-host transmission and public health risk should be interpreted cautiously. Therefore, despite the relatively low overall detection rate in black-headed gulls in this study, the zoonotic potential and public health risk associated with the ST7 subtype cannot be overlooked. Further surveillance of Blastocystis in migratory waterbirds and related environments would be useful to better assess its occurrence and potential transmission risk.

An interesting finding was the detection of the ST26 subtype (1/8 positives). ST26 is predominantly considered a ruminant-adapted lineage, commonly found in cattle and other ruminant species worldwide [35,36,37,38]. Previous studies have reported sporadic occurrences of this subtype in non-ruminant hosts, including mussels [39], oysters [2] and various natural water bodies [40,41]. Since these aquatic organisms and water environments serve as the primary food sources for black-headed gulls, we hypothesize that the gulls acquired infection via ingesting contaminated water or infected shellfish or mollusks. This phenomenon suggests that ST26 may have cross-species transmission potential with expanding host ranges. Furthermore, our findings highlight that *Blastocystis* harbored by aquatic organisms may pose a potential food-borne risk to public health. Accordingly, it is imperative to strengthen routine surveillance and implement targeted control strategies so as to guarantee food safety.

Although this study provided preliminary data on the fecal sample positivity and subtype composition of *Blastocystis* in wintering black-headed gulls in Kunming, several limitations still exist. First, this study only detected *Blastocystis* DNA in avian fecal samples. Although the fecal samples were collected immediately after direct observation of defecation by black-headed gulls, the results represent sample-level positivity rather than individual-level prevalence. In addition, fecal PCR detection alone cannot distinguish true intestinal colonization from transient mechanical passage of cysts acquired from contaminated water, sediment, or aquatic food sources. This limitation is particularly relevant to the single ST26-positive sample. Second, no supporting detection was performed on surrounding wetland water, sediment, aquatic organisms, humans, domestic animals, livestock, companion animals, or other wild birds in the same ecosystem. The lack of environmental and host source-tracing data makes it impossible to fully clarify the infection source, host sharing, cross-species transmission route, or contamination pathway of ST26 in black-headed gulls. Third, this study only completed molecular identification and phylogenetic analysis of *Blastocystis* subtypes based on the *SSU* rRNA gene. Multilocus genotyping, whole-genome sequencing, quantitative parasite load analysis, and viability or infectivity assays were not performed, which limited further understanding of genetic diversity, transmission pathways, and biological significance. No in vitro or in vivo pathogenicity verification experiments were conducted, and the actual pathogenic ability and intestinal damage mechanism of ST7 detected in black-headed gull fecal samples remain to be further verified. Finally, the sampling scope was restricted to Kunming Dianchi Lake during the wintering season, and multi-region comparative sampling was absent, which limits the universality and regional representativeness of the research conclusions. Because black-headed gulls occur in Kunming mainly as wintering migratory birds rather than resident birds, this study could not determine temporal variation, persistence of positivity, or changes along the migratory route. Future studies will expand the sampling duration and geographical scope, integrate environmental, human, and animal cross-sectional samples, apply higher-resolution molecular methods, and conduct functional verification of subtype pathogenicity to systematically reveal the epidemiological characteristics and potential transmission risk of *Blastocystis* in migratory birds.

## 5. Conclusions

This study reports a fecal sample positivity rate of 3.27% for Blastocystis in wintering black-headed gulls in Kunming, China. Two subtypes, ST7 and ST26, were identified, with ST7 being the most frequently detected subtype in this limited dataset. ST7 is a zoonotic subtype with pathogenic potential, whereas ST26, previously reported mainly in ruminants, is here detected in black-headed gulls for the first time globally. The detection of ST26 in one sample represents a preliminary finding that requires confirmation in larger populations and other locations. The presence of zoonotic subtypes in migratory black-headed gulls highlights the potential role of these birds in the cross-species transmission of Blastocystis and underscores the need for ongoing surveillance of migratory waterbirds as part of public health monitoring efforts.

## Figures and Tables

**Table 1:** Fecal sample positivity rate and subtypes of *Blastocystis* sp. in black-headed gulls (*Chroicocephalus ridibundus*) in Kunming, China.

Collection Date	Positive/Total Samples	Positive Rate (95% CI)	Subtype (No.)	*p*
2025.12.15	3/88	3.41% (0.46–7.28)	ST7 (3)	0.92
2026.01.16	5/157	3.18% (0.41–5.96)	ST7 (4), ST26 (1)	
Total	8/245	3.27% (1.02–5.51)	ST7 (7), ST26 (1)	

## Data Availability

The data presented in this study are openly available in GenBank, reference number PZ489957–PZ489964.
